# Coupling Fault Diagnosis of Bearings Based on Hypergraph Neural Network

**DOI:** 10.3390/s24196391

**Published:** 2024-10-02

**Authors:** Shenglong Wang, Xiaoxuan Jiao, Bo Jing, Jinxin Pan, Xiangzhen Meng, Yifeng Huang, Shaoting Pei

**Affiliations:** Aeronautics Engineering College, Air Force Engineering University, Xi’an 710038, China; phm_wsl@outlook.com (S.W.); jingbo_sensors@outlook.com (B.J.); panjinxin_sensors@outlook.com (J.P.); mengxz_sensor@outlook.com (X.M.); huangyiff@189.cn (Y.H.); pst_phm@outlook.com (S.P.)

**Keywords:** coupling fault diagnosis, feature generation, feature extraction, hypergraph networks

## Abstract

Coupling faults that simultaneously occur during the operation of mechanical equipment are widespread. These faults encompass a diverse range of high-order coupling relationships, involving multiple base fault types. Based on the advantages of hypergraphs for higher-order relationship descriptions, two coupling fault diagnosis architectures based on the hypergraph neural network are proposed in this paper: 1. In the coupling fault diagnosis framework based on feature generation, the base faults serve as the hypergraph nodes, and each hyperedge connects the base faults. The generator, which consists of the hypergraph neural network, generates coupling faults as negative samples to enforce regularization constraints for the discriminator training. 2. In the coupling fault diagnosis framework based on feature extraction, each node represents a fault mode, and each hyperedge connects nodes with common failure modes. The multi-head attention mechanism extracts the features of base faults, and the common fault features in a hyperedge are aggregated via the hypergraph neural network. The inner product correlation is used to diagnose the fault modes. The results show that the diagnostic accuracy for coupling faults with the two frameworks reaches 88.6% and 86.76%, respectively. Both frameworks can be used for the diagnosis and analysis of high-order coupling faults.

## 1. Introduction

Coupling faults are widespread in mechanical equipment. They are formed via the coupling of multiple types of base faults. Coupling faults are not linear superpositions of base faults, and complex fault characteristics emerge in the coupling process [1]. Therefore, simple linear decoupling cannot accurately diagnose coupling faults, and a more accurate nonlinear model needs to be established to describe the fault coupling process [2]. In this study, two kinds of data-driven methods are used to obtain the fault characteristics of multi-fault coupling, reveal the fault coupling process, and classify the coupling faults based on the obtained coupling fault characteristics.

The fault characteristics of rotating machinery can be obtained using spectral analysis [3]. Scholars have mostly used traditional frequency domain feature extraction methods [4]. Ma et al. [5] proposed adaptive demodulation analysis to extract early bearing fault features. Jiang et al. [6] used a short-time Fourier transform to obtain the bearing amplitude and used adaptive weights to extract the weighted characteristic frequency. Zhu et al. [7] determined the hyperparameters of the spectral amplitude modulation (SAM) model by taking the maximum harmonic significance as an index. They divided the frequency band and reconstructed the signal to suppress the noise component in the fault characteristics. Frequency domain features analyze all frequency bands in the spectrum, and the energy of fault features is not concentrated. Several scholars have used time–frequency domain feature extraction methods to extract the fault features of energy-concentrated frequency bands [8]. Yue et al. [9] proposed multi-scale wavelet networks to extract multi-scale features from vibration signals and used meta-learning modules to learn the distance distribution between data to achieve model generalization. This method establishes a distance-based data space, but the definition of distance lacks explainability. Tang et al. [10] used a time–frequency domain convolutional neural network to extract the fault features of multiple energy concentrations and introduced an attention module to extract weighted representative features. Jia et al. [11] proposed a Gramian-based noise reduction strategy for noise suppression and feature extraction. Yoo et al. [12] carried out convolutional neural network (CNN) image processing based on a spectrum diagram to extract the fault features in an image. These methods extract the features of a single fault from the spectrum, but there is no effective solution to extract the features of the coupled fault. Based on the prior fault information [13], the extracted feature acquisition method decouples the fault coupling process and fits the basic fault correlation weight, which belongs to the Bayes school category.

The data-driven fault feature generation method can avoid the difficulty of spectrum extraction caused by the lack of working condition information [14]. Zhang et al. [15] generated fault features adaptively via convolutional neural networks and used support vector machine (SVM) classifiers to classify the fault types. Karnavas et al. [16] proposed using two attention mechanisms to extract global and local features, respectively, and fuse these two types of features. Sivapriya et al. [17] used multi-scale and different resolution convolution check fault information to extract and fuse it into global fault features for fault diagnosis. Yang et al. [18] reconstructed fault features via IMFs extracted from variational mode decomposition (VMD) and used SVMs for fault classification given the unbalanced and strong noise characteristics of bearing signals. Huo et al. [19] carried out the adaptive extraction of time–frequency domain features and combined harmonic detection with noise signals to continue reconstructing the features. Zhao et al. [20] reduced the dimensionality of features using depth-separable convolutional blocks and used the global feature awareness module to perform adaptive weighting processing similar to the attention mechanism for the feature signal channels to obtain the fault features. Yang et al. [21] reconstructed coupling fault features using an autoencoder to achieve feature dimension reduction and non-equilibrium data strengthening. Jang et al. [22] combined an adversarial network based on an autoencoder to generate the original data stream. Most of these generative methods reconstruct the original data, but the reconstructed data lack interpretability. Yu et al. [23] proposed a zero-sample fault diagnosis method. The digital twin method was used to generate fault data based on the health state model. The data space generated with this method was uncontrollable and could not achieve accurate fault classification. The generative feature acquisition method reconstructs the critical fault information from the existing fault information and estimates the overall probability by observing many samples; this belongs to the frequency school.

Both extraction and generative methods obtain the fault characteristics of independent bearing faults [24]. Non-Euclidean data-oriented fault diagnosis methods should be used for complex coupling faults that are widespread in bearings [25,26]. A graph neural network constructs the topological structure of the data via the nodes and the edges connecting the nodes, which can realize the correlation description of non-Euclidean data. A traditional graph neural network describes the relationship between node pairs [27]. Some scholars have used graph neural networks to extract fault features and perform fault diagnosis. Li et al. [28] established a graph convolutional network (GCN) with multiple receptive fields to conduct adaptive correlation analysis on vibration data after FFT and took data with different receptive fields as the fault characteristics. Wang et al. [29] used a bidirectional graph neural network to carry out correlation analysis of the graph data extracted from a residual network at the instance and distribution levels. This classified independent faults, but the network needs to be further optimized for correlation analysis between coupled faults. Wang et al. [30] added dynamic vertices to a graph neural network and realized coupling fault diagnosis by establishing a pair relationship topology between faults and dynamic vertices.

It is difficult for graph neural networks to achieve high-level expressions of the relationships between objects [31]. Feng et al. [32] proposed a hypergraph neural network (HGNN) to solve this problem. Bai et al. [33] introduced convolution and attention mechanisms into hypergraph neural networks, further improving the ability of representation learning in hypergraph networks. Hypergraph neural networks are mainly used in recommendation systems and multi-modal data feature extraction and have been widely used in image processing. Shi et al. [34] built unlabeled data into hypergraphs, mined the data’s higher-order information, and combined an autoencoder to realize representation learning and fault classification. In this method, the hypergraph structure is taken as an autoencoder layer, and the fault features are generated from the data-driven perspective, which lacks interpretability. Su et al. [35] extracted the higher-order relationship between the same type of faults and different types of faults via the hypergraph structure. These methods are applied to independent fault diagnosis. In the field of coupled fault diagnosis, the high-order relation representation ability of hypergraph neural networks meets the requirement of complex coupled fault feature acquisition. In this paper, two fault diagnosis architectures based on hypergraph neural networks are proposed: a coupled fault diagnosis architecture based on feature generation and another based on feature extraction. The method is verified with coupling fault injection experimental data.

The main insights and contributions of this study are summarized as follows:

(a) A rotary mechanical coupling fault injection test is designed. Eight types of faults including coupling faults and independent faults are injected, and the experimental data are collected.
(b) A coupled fault diagnosis architecture based on feature extraction is proposed. A hypergraph generative adversarial network (HGGAN) is established and vibration data generation and coupling fault diagnosis are realized by applying hypergraph theory to the generative adversarial network (GAN) model.
(c) A coupled fault diagnosis architecture based on feature generation is proposed. The coupling fault characteristics are extracted via a multi-head inner product hypergraph attention network (IPHGAT), and coupling fault diagnosis and analysis are realized.

The rest of this paper is organized as follows: Section 2 introduces the preliminary knowledge, including the graph attention network, the hypergraph attention network, and the generative adversarial network. Section 3 introduces the two fault diagnosis frameworks based on a hypergraph attention network. The coupled fault injection experiment and data-preprocessing methods are introduced in Section 4. The two coupled fault diagnosis frameworks are conducted in Section 5. Finally, conclusions are drawn in Section 6.

## 2. Preliminary Knowledge

### 2.1. Graph Attention Network

In graph G=(V,E), E is the set of edges, and V is the set of nodes. In the GCN, one step of graph convolution is defined as follows [36]:

(1) $$X^{l+1}=\sigma \left({\tilde{D}}^{-\frac{1}{2}}A{\tilde{D}}^{-\frac{1}{2}}X^{l}W^{l}\right)$$

where A is the adjacency matrix, $\tilde{D}$ is the degree matrix with a self-loop, and W is the linear transformation weight matrix. The graph attention network (GAT) adds the attention mechanism into the graph convolutional network and uses the dynamic adjacency matrix to perform the adaptive values of the edge vector according to the node data characteristics. The calculation method is as follows [37]:

(2) $$A_{ij}=\frac{\exp\left(\;\mathrm{LeakyReLU}\;\left(a^{T}\left[h_{i}W\|h_{j}W\right]\right)\right)}{\sum_{k\in N_{i}}\exp\left(LeakyReLU\left(a^{T}\left[h_{i}W\|h_{k}W\right]\right)\right)}\;$$

The GAT only describes the dynamic edges between pairwise nodes. Higher-order representation methods are needed for modeling processing for the multiple interactions in coupling faults.

### 2.2. Hypergraph Attention Network

The hypergraph convolutional network (HGCN) connects multiple nodes via hyperedges, which extract higher-order relationships between the nodes. The inter-layer information transmission mode is as follows:

(3) $$X^{l+1}=D_{v}^{-1/2}AHD_{e}^{-1}A^{T}D_{v}^{-1/2}X^{l}W^{l}$$

where A is the adjacency matrix, $D_{v}$ is the node degree matrix, $D_{e}$ is the hyperedge degree matrix, H is the weight diagonal matrix of the hyperedge, and W is the linear transformation weight matrix. The hypergraph attention network (HGAT) computes the values in the adjacency matrix A, and the correlations between nodes are quantitatively described. The computation of the attention mechanism in the hypergraph structure is defined as follows:

(4) $$A_{ij}=\frac{\exp\left(\mathrm{LeakyReLU}(a^{T}[x_{i}W\|e_{j}W])\right)}{\sum_{k\in N_{i}}\exp\left(\mathrm{LeakyReLU}(a^{T}[x_{i}W\|e_{k}W])\right)}$$

where $N_{i}$ is the neighborhood set of $x_{i}$, [,∥,] is the concatenation operation, and a is the weight vector. The similarity of the two vectors can be acquired after concatenation calculation.

The attention score in the GAT describes the correlation between a pair of nodes, while the attention score in the HGAT is the correlation between nodes and hyperedges. Therefore, applying an attention mechanism in hypergraphs requires nodes and hyperedges to be in the same homogeneous domain.

### 2.3. Generative Adversarial Network

The adversarial generation network models the data probability distribution via the game between the generator model G(z) and discriminator model D(x) [38]. The input of the generator is a random vector z, and the loss function of the generator model is as follows:

(5) $$Loss_{G}=\frac{1}{M_{fake}}\sum_{i=1}^{M_{fake}}\log(1-D(G(z^{\left(i\right)})))$$

where $M_{fake}$ is the number of fake data samples. The input of the discriminator is x, and the loss function is as follows:

(6) $$Loss_{D}=-\frac{1}{S}\sum_{i=1}^{M_{real}}y_{real}^{i}\log(D(x^{i}))-\frac{1}{S}\sum_{i=1}^{M_{fake}}\left(1-y_{fake}^{i})\log(1-D(G(z^{i}))\right)$$

where $S=M_{real}+M_{fake}$ is the total number of false samples and true samples, and $M_{real}$ is the number of true samples. The first half of the loss function requires the discriminator to be true to the real data, and the second half requires the discriminator to be false to the false data.

## 3. Algorithm Flow

Coupling faults are formed by coupling multiple types of base faults [39], which interact with each other. Therefore, the fault characteristics of coupling faults are not simple linear superpositions of base faults but nonlinear complex coupling. In this study, the vibration characteristics of coupled faults are not decomposed from the complex physical mechanism, but the advantages of the hypergraph neural network in processing higher-order relationships are used to model the coupling faults.

### 3.1. Algorithm Flow of HGGAN

From the perspective of holism, the coupling faults of mechanical equipment comprise many kinds of faults, which show unified fault characteristics. Assuming that the fault signals of coupled faults are generated by the implicit nonlinear coupling between the fault signals of the base faults, this study uses a hypergraph neural network to model the nonlinear generation process and form the coupled fault diagnosis model, HGGAN. In this model, the graph node set V is composed of M, many base faults, i.e., $\left|V\right|=M$. Each base fault and coupling fault constitute N, many hyperedges, i.e., $\left|E\right|=N$, where M≤N, which is reduced to equality if, and only if, all nodes are base faults.

**Figure 1:**
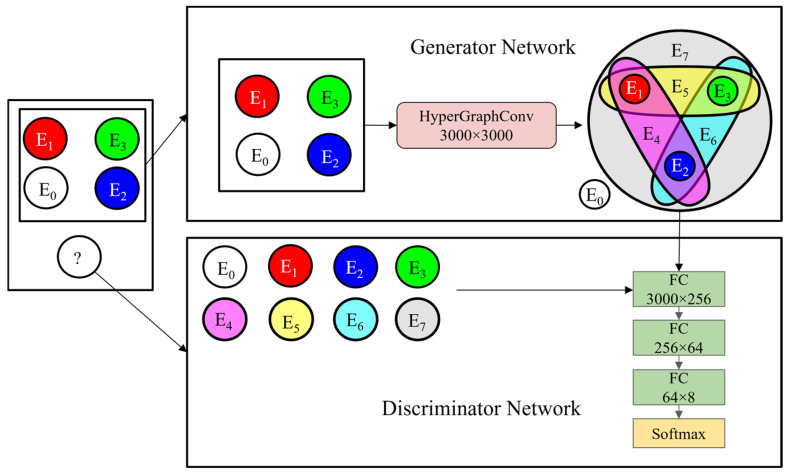
HGGAN structure.

A coupled fault diagnosis architecture based on feature generation is designed in this study based on the ability of the GAN to generate a probability distribution of the data. The generator consists of the HGCN, and the discriminator is composed of a multi-layer perceptron (MLP). The algorithm flow is shown in Figure 1.

In the HGGAN model proposed in this paper, the generator’s loss function is rewritten:

(7) $$Loss_{G}=\frac{1}{S}\sum_{i=1}^{S}\sum_{j=1}^{M}\log(1-D(G(x_{j}^{i})))$$

The loss of the generator is the classification error of the fault data coupled with the base fault, where S is the total number of samples. Compared with the traditional GAN, the input of the generator changes from a random vector z to the fault feature x of the base fault in the graph data $x_{j},j\in 1,\cdots ,M$, which improves the model’s stability compared with random data [40]. Both true and false samples come from the same graph data sample, so the numbers of true samples and false samples are equal.

The loss function of the discriminator is rewritten as follows:

(8) $$Loss_{D}=-\frac{1}{S}\sum_{i=1}^{S}y_{M+1}^{i}\log(D(x_{M+1}^{i}))-\frac{\alpha }{S}\sum_{i=1}^{S}\sum_{j=1}^{M}\left(1-y_{j}^{i})\log(1-D(G(x_{j}^{i}))\right)$$

The rewritten discriminator loss function is still composed of two parts. In the first part, the input $x_{M+1}$ of the discriminator is the dynamic vertex in the graph data, that is, the fault feature of the coupling fault to be diagnosed, and its label $y_{M+1}$ is the fault type corresponding to the coupling fault $x_{M+1}$. The second part is the regularization term to prevent the model from overfitting, where the hyperparameter α is set as 0.1 in this article.

The HGGAN established in this paper generates coupling fault features from the perspective of data generation. A hypergraph convolution network is used as the generator, which avoids the uncertainty caused by random vectors in a traditional GAN and provides the network structure with interpretability.

### 3.2. Algorithm Flow of IPHGAT

From the perspective of reductionism, the coupling faults of mechanical equipment comprise multiple base faults. The signals of coupling faults can be decomposed into those belonging to the base faults. In this framework, a coupling fault diagnosis model based on IPHGAT is established. All the fault modes, including base faults and coupling faults, are taken as graph nodes, and $\left|V\right|=M$. Hyperedges are the fault characteristics of all fault modes after aggregation, and $\left|E\right|=N$. The number of nodes and hyperedges are equal, i.e., M=N. Therefore, this study applies an attention mechanism to further analyze and process the fault information. The correlation between each base fault and each type of fault is obtained via the attention mechanism.

In the multi-head attention mechanism, the number of heads is defined as the number of independent base failures, and the multi-head attention scores are calculated as follows:

(9) $$A_{ij}^{k}=\frac{\exp\left(\mathrm{LeakyReLU}(a^{k^{T}}[x_{i}W^{k}\|e_{j}W^{k}])\right)}{\sum_{n\in N_{i}}\exp\left(\mathrm{LeakyReLU}(a^{k^{T}}[x_{i}W^{k}\|e_{n}W^{k}])\right)}$$

where x is the node vector, and e is the hyperedge vector. The trainable parameter $a^{k}$ is the direction of the attention calculation corresponding to the k-th head. The output of the l-th layer network is calculated according to the multi-head attention extraction features:

(10) $$x_{j}^{l+1}=\sigma \left({\|}_{k=1}^{m}\frac{1}{\left|N_{j}\right|}\sum_{i\in N_{j}}\alpha_{ij}^{l,k}W^{l}x_{i}^{l}\right)$$

In the hypergraph attention mechanism proposed in this paper, the updated coupled fault node vector is used to update the hyperedge vector, i.e., $e_{j}^{l+1}=x_{j}^{l}$.

**Figure 2:**
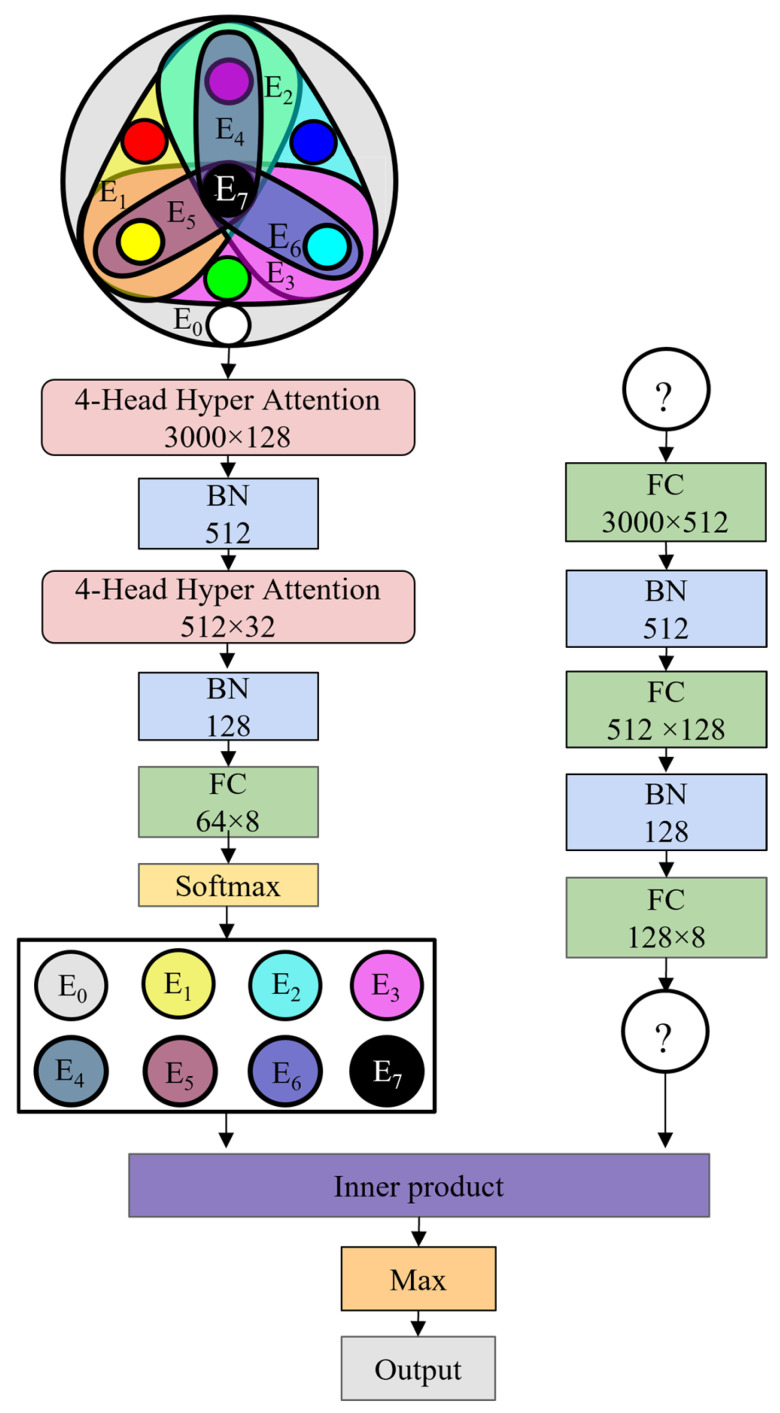
IPHGAT network structure.

The network structure is shown in Figure 2. The algorithm flow of the IPHGAT model is as follows.

Step 1: The constructed hypergraph data $X=[X^{\left(0\right)},X^{\left(1\right)}]\in {\mathbb{R}}^{(M+1)\times Q}$ are inputted, where Q is the data feature dimension, and X includes the M base fault node data $X^{\left(0\right)}\in {\mathbb{R}}^{M\times Q}$ and the fault $X^{\left(1\right)}\in {\mathbb{R}}^{Q}$ to be predicted located at a dynamic vertex. The corresponding labels are $Y^{\left(0\right)}=[1,\cdots ,M]$, $Y^{\left(1\right)}\in R$. The $X^{\left(0\right)}$ passes through the first layer of m−head HGAT, generates m features for each hyperedge, splices the m features of each hyperedge into a whole as the features of the hypergraph, and performs feature standardization to obtain $X_{1}^{\left(0\right)}$.

Step 2: $X_{1}^{\left(0\right)}$ is inputted into the second layer of m−head HGAT to obtain $X_{2}^{\left(0\right)}$, and then input $X_{2}^{\left(0\right)}$ into the fully connected network and Softmax layer to obtain the fault feature ${\hat{Y}}^{\left(0\right)}\in {\mathbb{R}}^{m\times m}$ corresponding to each hyperedge. The cross-entropy of ${\hat{Y}}^{\left(0\right)}$ and $Y^{\left(0\right)}$ is regarded as the loss function $L_{1}$.

Step 3: $X^{\left(1\right)}$ is inputted into the three-layer MLP to obtain the fault feature ${\hat{Y}}^{\left(1\right)}\in {\mathbb{R}}^{m}$ of the node to be predicted. The cross-entropy of ${\hat{Y}}^{\left(1\right)}$ and $Y^{\left(1\right)}$ is set as the loss function $L_{2}$.

Step 4: The inner product between the vector ${\hat{Y}}^{\left(1\right)}$ and ${\hat{Y}}^{\left(0\right)}$ corresponding to each hyperedge is calculated as $C=[c_{1},\cdots ,c_{M}]$, the label $\hat{Y}$ of the hyperedge corresponding to the vector with the largest inner product value is taken, and the model accuracy rate is obtained by judging whether $\hat{Y}$ and ${\hat{Y}}^{\left(1\right)}$ are equal.

The loss function of the model consists of two parts, $L=L_{1}+L_{2}$, where $L_{1}=CrossEntropy(Y^{\left(0\right)},{\hat{Y}}^{\left(0\right)})$ and $L_{2}=CrossEntropy(Y^{\left(1\right)},{\hat{Y}}^{\left(1\right)})$. The value of the corresponding fault mode’s hyperedge vector tends to increase via the model training process, thus driving the inner product to increase.

## 4. Data Acquisition and Preprocessing

**Figure 3:**
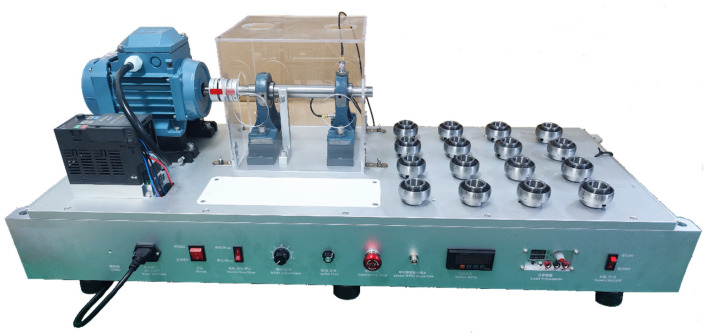
Coupling fault test bench.

**Figure 4:**
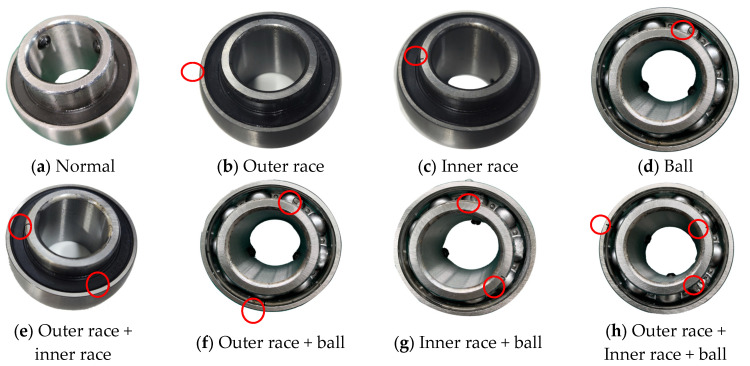
Coupling fault injection bearing.

In the experiments in this study, 8 faults were injected into the UCPH205 bearing. The test bench is shown in Figure 3. The samples included 1 normal bearing, 4 outer-race fault bearings, 4 inner-race fault bearings, 4 ball fault bearings, 1 outer-race + inner-race fault bearing, 1 outer-race + ball fault bearing, 1 inner-race + ball fault bearing, 1 outer-race + inner-race + ball fault bearing, and 1 outer-race + inner-race + ball fault bearing, as shown in Figure 4. The fault injection experiment was carried out at 2400 rpm for each bearing. A sample rate of 20 kHz was used to collect the experimental data, and the fault size of the outer race, the inner race, and the ball was 0.3 mm.

**Figure 5:**
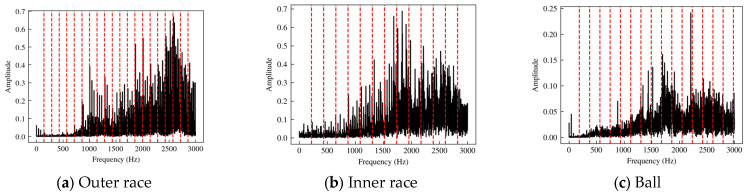
Spectra of base fault bearings.

Each bearing ran for 60 s at different speeds. The data for each second were taken as a sample, containing a total of 1020 samples. In this study, the *X*-axis vibration signal was selected for fault diagnosis and analysis. Firstly, the Fourier transform was performed on the data to obtain the spectra of the vibration data. The fault spectra of the base faults are shown in Figure 5.

The red dashed line is the fundamental frequency. Its frequency—double that of the fault characteristic frequency value—was calculated theoretically based on the bearing size, in which the fundamental frequencies of the outer-race, inner-race, and ball faults were 142.93 Hz, 217.07 Hz, and 186.27 Hz, respectively. The figure shows that the fault frequency of the bearing-injected fault had a high consistency with its theoretical value.

**Figure 6:**
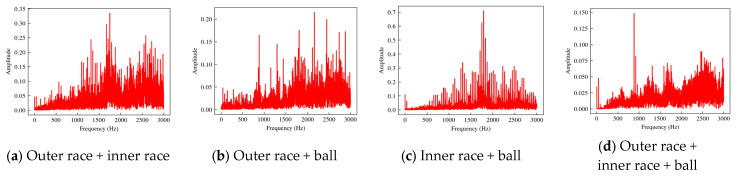
Spectra of coupling fault bearings.

The spectra of the coupled fault data are shown in Figure 6. The vibration spectrum of a coupled fault is not a linear superposition of the base fault spectrum but contains complex coupling relations, which requires a higher-order relationship model to obtain the coupling fault features.

## 5. Coupling Fault Diagnosis

### 5.1. Coupling Fault Diagnosis Based on Feature Generation

**Figure 7:**
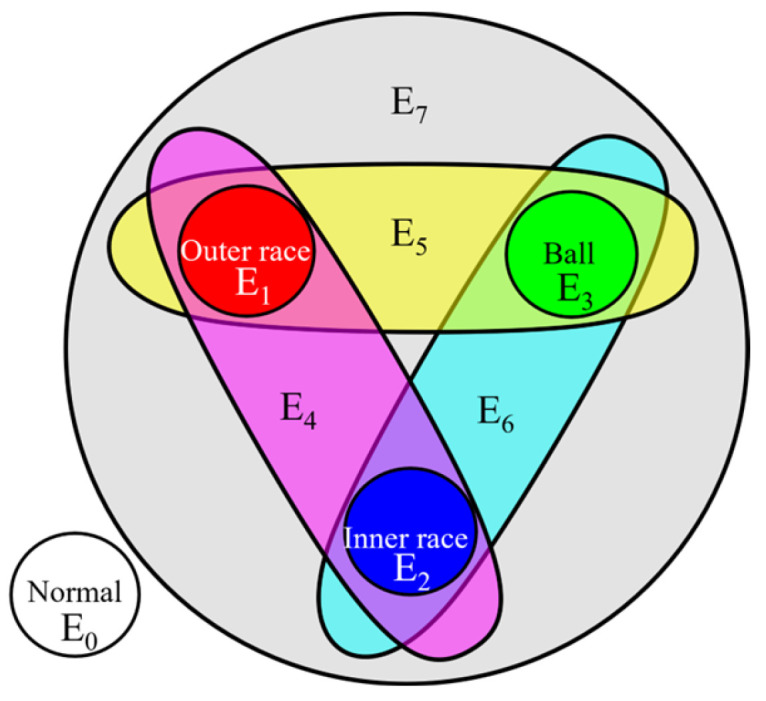
Coupled fault topology in HGGAN.

In the HGGAN architecture, the four base faults are defined as four graph nodes, and the eight hyperedges represent eight types of coupling faults. As shown in Figure 7, the normal state constitutes an independent hyperedge; the inner-race, the outer-race, and the ball faults each constitute an independent hyperedge, in three primary colors; the pair–pair couplings constitute three hyperedges; and the three-fault coupling constitutes hyperedge $E_{7}$, comprising a total of eight hyperedges. The incidence matrix is shown in Table 1.

**Figure 8:**
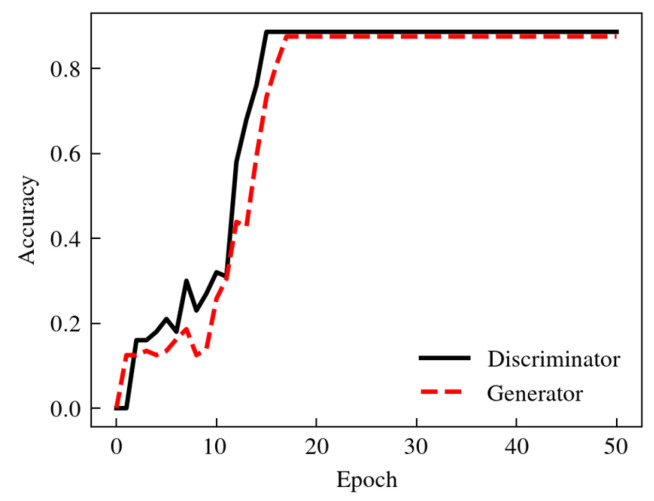
Accuracies of discriminator and generator during training process of HGGAN.

The dataset was constructed based on the hypergraph topology, in which the training set contained 816 hypergraph samples, and the test set contained 204 hypergraph samples. The SGD optimizer was used for model training, the SGD momentum was set to 0.9, the step dynamic learning rate was adopted, the initial learning rate was 0.01, and the decay rate was 0.1. The training results are shown in Figure 8.

The accuracies of the discriminator and generator were 88.6% and 87.5%, respectively. The discriminator and generator played games with each other in the training process to jointly optimize the model’s accuracy.

**Figure 9:**
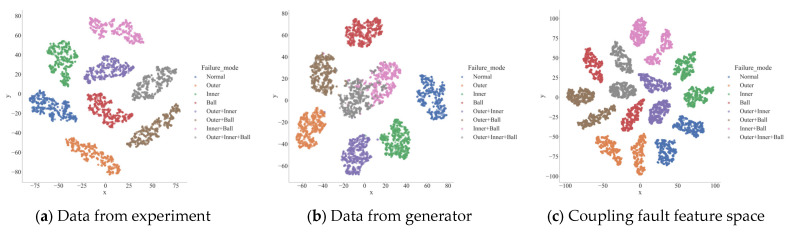
Visualization of data from experiment and data generated by generator.

The generator aggregated the three types of base faults via the hypergraph network to obtain a total of $2^{3}=8$ types of faults. The experimental data and the data generated by the generator were reduced to two dimensions by the TSNE, and their distributions are shown in Figure 9. As shown in Figure 9a, the experimental data were separated after preprocessing in the feature space, but the coupling effect of the fault was not displayed in the feature space. The output data features of the generator are shown in Figure 9b, where the third-order coupling fault represented by the gray data points is approximately located in the fault center, and the second-order fault is located between the two base faults that compose it. Although there was a clear dividing line between the data generated by the generator and the original data, similar faults were approximately located in the same area in the two-dimensional plane, as shown in Figure 9c. The reason is that the generator loss function is the cross-entropy of the network output and the real label, which only has requirements for the size relationship of the output value and has low requirements for the consistency of data values so that the model has good generalization. Therefore, the data generated by the generator can realize more accurate fault diagnosis.

The discriminator obtained by the HGGAN model was compared with the MLP without adding a generator and the MLP-GAN model in which the MLP was both the generator and discriminator. Moreover, it was compared with the model with a residual in the discriminator. The diagnostic accuracies are shown in Table 2. The HGGAN had advantages over the other models in terms of accuracy.

Compared with the MLP, the HGGAN’s discriminator had the same structure as the MLP. The HGGAN’s loss function was optimized by adding the generator to HGCN to generate negative samples and optimize the MLP model parameters, so its fault diagnosis accuracy was improved. Compared with the MLP-GAN, the HGGAN used the HGCN as a generator to extract higher-order relationships more effectively and generate more accurate fault feature data. The shortcut was added to the discriminator to introduce the residual block. The residual block blurred the extracted features because of the shallow network depth, resulting in a lower discriminator accuracy. There was no improvement in the generator performance compared with the HGGAN proposed in this article.

### 5.2. Coupling Fault Diagnosis Based on Feature Extraction

**Figure 10:**
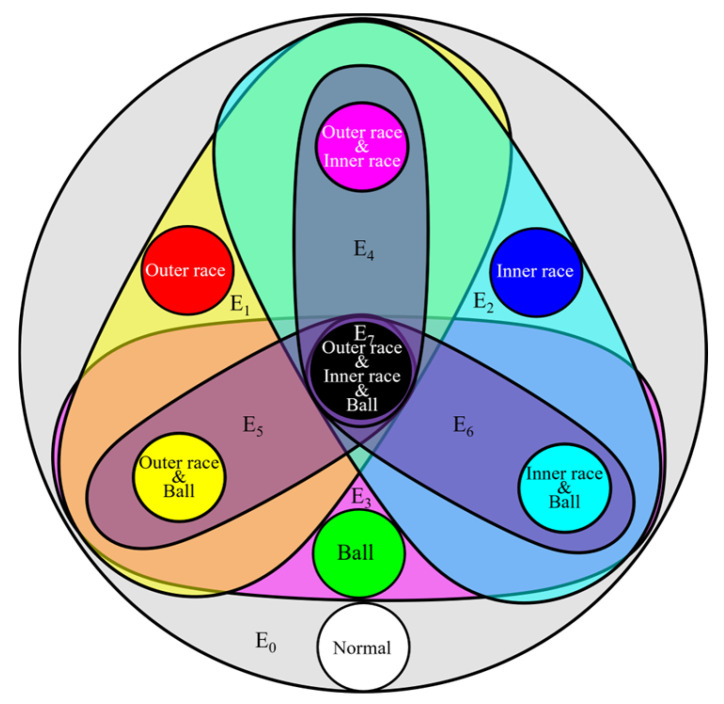
Coupled fault topology in IPHGAT.

This study considered four base faults in the IPHGAT architecture—normal, outer-race, inner-race, and ball faults—and four coupled faults—outer-race + inner-race, outer-race + ball, inner-race + ball, and outer-race + inner-race + ball faults—as the graph nodes and established eight hyperedges corresponding to all fault types. The hypergraph topology is shown in Figure 10.

The correlation matrix of the hypergraph topology was obtained according to the fault coupling relationship, as shown in Table 3.

The hypergraph data $X=[X^{\left(0\right)},X^{\left(1\right)}]\in R^{9\times 3000}$ were built for training. The SGD optimizer was used for model training, the SGD momentum was set to 0.9, a step dynamic learning rate was adopted, the initial learning rate was 0.01, and the attenuation rate was 0.1. Since the number of base faults involved in this study was four, the number of heads in the multi-head IPHGAT was set to four.

**Figure 11:**
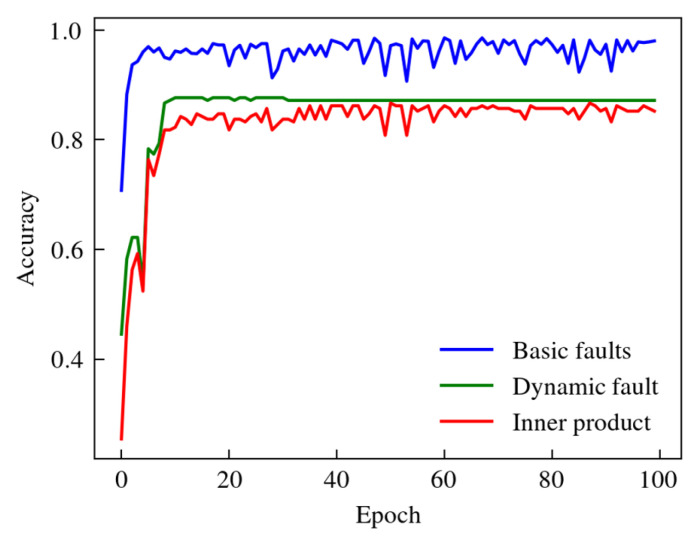
Diagnostic accuracy for base faults, faults in dynamic vertex, and inner product during training process of IPGAT.

In the training process, the classification accuracy $Acc^{\left(0\right)}$ of the base fault $X^{\left(0\right)}$, the accuracy $Acc^{\left(1\right)}$ of the dynamic vertex fault $X^{\left(1\right)}$ to be predicted, and the correlation accuracy Acc of the inner product were calculated. As shown in Figure 11, $Acc^{\left(0\right)}$ and $Acc^{\left(1\right)}$ reached 99.88% and 88.60%, respectively, and the internal product correlation accuracy rate Acc reached 86.76%, so the IPHGAT can effectively classify coupling faults.

Table 4 compares the coupled fault diagnostic accuracies of the three methods. The accuracy reached 78.43% by adding a residual network to the model, failing to improve the accuracy. The HGCN was used to extract coupled fault features, and the accuracy of its training set reached 88.6%, consistent with that of the IPHGAT with the MLP proposed in this paper. However, only global fault features were extracted due to its lack of local feature extraction capability. Furthermore, its generalization was poor, reaching only 83.82% accuracy on the test set.

The IPHGAT contained two layers of four-head hypergraph attention networks, which were used for the feature extraction of four types of basic faults. Each sample included eight types of faults, and the eight elements in the output ${\hat{Y}}^{\left(0\right)}\in {\mathbb{R}}^{8\times 8}$ of the Softmax layer corresponded to eight types of fault modes. The first four elements ${\hat{Y}}_{i}^{\left(0\right)}=\{{\hat{y}}_{i,1},{\hat{y}}_{i,2},{\hat{y}}_{i,3},{\hat{y}}_{i,4}\}$ were the correlation between the four base faults and the fault mode corresponding to the row vector $x_{i}$. The four base fault correlation elements corresponding to the eight types of faults in each sample were extracted, and the coupling mode of the faults was analyzed.

The model parameter optimization aimed to improve the fault classification accuracy during the model training. ${\hat{y}}_{i,i}$ is generally the maximum value in ${\hat{Y}}^{\left(0\right)}$ and is at a higher order of magnitude than other elements, so this study carried out normalization processing on ${\hat{y}}_{i,1},{\hat{y}}_{i,2},{\hat{y}}_{i,3},{\hat{y}}_{i,4}$ as follows:

(11) $${\bar{y}}_{i,j}=\frac{{\hat{y}}_{i,j}-\min({\hat{Y}}_{i}^{\left(0\right)})}{\max({\hat{Y}}_{i}^{\left(0\right)})-\min({\hat{Y}}_{i}^{\left(0\right)})}$$

**Figure 12:**
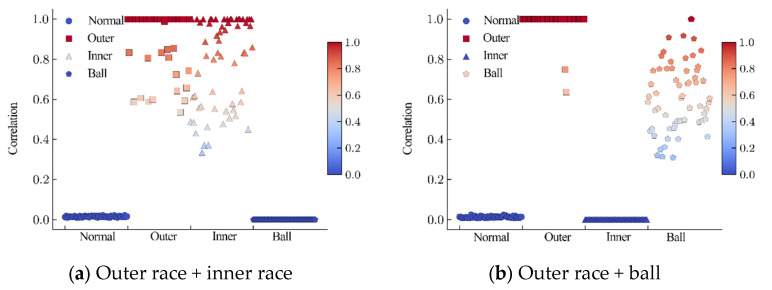

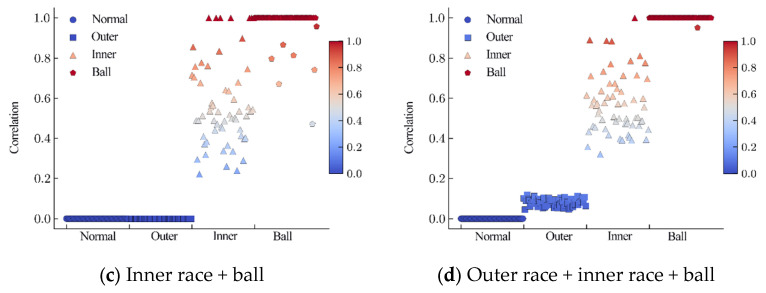
Base fault correlations.

Since the model achieved accurate feature extraction of the four types of independent base faults, a correlation analysis between the base faults and coupled faults was carried out, as shown in Figure 12. As shown in Figure 12a–c, the base faults for the second-order coupling faults were effectively identified, and the correlations between the coupling faults and the unrelated base faults were suppressed. In the base fault correlation analysis of the third-order coupling faults, all three types of base faults showed correlations with the coupled faults, and the correlation between the normal state and coupled faults approached zero, as shown in Figure 12d.

**Figure 13:**
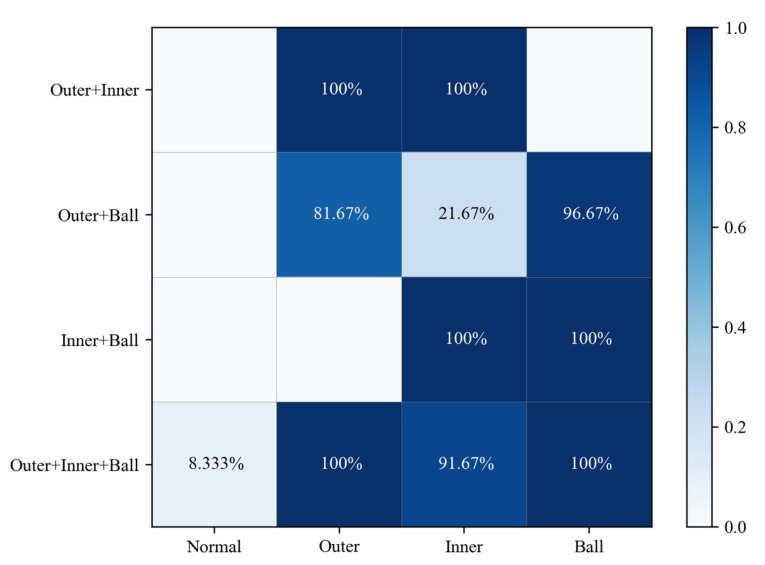
Coupling fault classification accuracies.

The inner product correlation between the dynamic vertex faults and eight types of fault data was $C=[c_{1},\cdots ,c_{8}]\in R^{8}$. The inner product correlation $c_{1},c_{2},c_{3},c_{4}$ of the coupled fault and base fault characteristics was removed for the analysis in Figure 13. The vertical axis is the coupling fault to be predicted, and the horizontal axis is the base fault that makes up the coupling fault. The accuracy of the base fault components of the coupled faults was more than 91.67%. The coupling analysis was consistent with the fault injection model.

The IPHGAT model based on the reductionist feature extraction framework extracts the fault component representing the base fault from the coupling fault and analyzes the fault mode based on the correlation of the fault component, which enhances the model’s interpretability and is significant for the coupling fault diagnosis of rotating machinery in industrial production environments.

## 6. Discussion and Conclusions

In this study, based on the research on the coupling fault diagnosis of rotating machinery, a coupling fault diagnosis method, HGGAN, based on a fault feature generation framework, and another method, IPHGAT, based on a fault feature extraction framework, were proposed from the perspectives of holism and reductionism theory by utilizing the advantages of a hypergraph neural network for higher-order relation analysis. The HGGAN used an MLP as a discriminator and utilized an HGCN as a generator to generate coupling fault data. The generated and real data had a unified data space, and the diagnostic accuracy of the HGGAN for coupling faults reached 88.6%. IPHGAT used a multi-head hypergraph neural network to extract the fault features of coupling faults, and an MLP was used to extract the dimensionality reduction features of predictive data. The internal product correlation between the fault features and coupling faults in the dynamic vertex was calculated, and the fault diagnosis of coupling faults was realized. The diagnostic accuracy rate reached 86.76%. The two kinds of methods have their own advantages in application. The HGGAN takes the generator error as the regularization term of the discriminator, which improves its diagnostic accuracy, which is slightly higher than that of IPHGAT. IPHGAT can evaluate the correlation between coupling faults and base faults based on the local characteristics of the base faults; thus, IPHGAT has higher interpretability than the HGGAN model.

The coupling fault diagnosis framework proposed in this paper can accurately diagnose coupling faults under high-order relations and analyze the coupling characteristics of the fault characteristics, which is significant for monitoring and analyzing the health state of rotating machinery in production environments. This study qualitatively analyzed the types of base faults that constituted the coupling faults in the interpretability analysis of the model. However, it did not verify the quantitative relationship between the correlation of the base faults and coupling faults extracted by the model and the degree of faults. In the next stage of coupled fault diagnosis research, multi-degree coupled fault injection tests should be carried out to further fit the quantitative relationship of the fault correlation with several fault samples.

## Figures and Tables

**Table 1:** Incidence matrix of HGGAN.

Fault Types	$E_{0}$	$E_{1}$	$E_{2}$	$E_{3}$	$E_{4}$	$E_{5}$	$E_{6}$	$E_{7}$
Normal	1	0	0	0	0	0	0	0
Outer race	0	1	0	0	1	1	0	1
Inner race	0	0	1	0	1	0	1	1
Ball	0	0	0	1	0	1	1	1

**Table 2:** Coupled fault diagnosis accuracies under feature generation framework.

Model	Accuracy of Generator	Accuracy of Discriminator
MLP	/	86.27%
MLP-GAN	75%	86.27%
Residual HGAN	87.5%	86.27%
HGGAN	87.5%	88.6%

**Table 3:** Comparison of diagnostic accuracy for coupling faults.

Fault Type	$E_{0}$	$E_{1}$	$E_{2}$	$E_{3}$	$E_{4}$	$E_{5}$	$E_{6}$	$E_{7}$
Normal	1	0	0	0	0	0	0	0
Outer race	1	1	0	0	0	0	0	0
Inner race	1	0	1	0	0	0	0	0
Ball	1	0	0	1	0	0	0	0
Outer race + inner race	1	1	1	0	1	0	0	0
Outer race + ball	1	1	0	1	0	1	0	0
Inner race + ball	1	0	1	1	0	0	1	0
Outer race + inner race + ball	1	1	1	1	1	1	1	1

**Table 4:** Coupled fault diagnostic accuracy under feature extraction framework.

Model	Training Set Accuracy	Test Set Accuracy
IPHGAT (residual)	83.7%	78.43%
IPHGCN	88.6%	83.82%
IPHGAT (MLP)	88.6%	86.76%

## Data Availability

The data are unavailable due to privacy.
